# The Impacts of End‐of‐Life Care Stress, Perception of a Good Death, and Emotional Intelligence on End‐of‐Life Care Performance Among Public Hospital Nurses

**DOI:** 10.1155/jonm/4401211

**Published:** 2026-08-23

**Authors:** Mi Gyoung Kim, Saekyae Shin, Minkyung Gu, Sohyune Sok

**Affiliations:** ^1^ Department of Nursing, Graduate School, Kyung Hee University, Seoul, Republic of Korea, khu.ac.kr; ^2^ Department of Nursing Science, College of Science, Kyungsung University, Busan, Republic of Korea, ks.ac.kr; ^3^ Department of Nursing, College of Health Science, Daejin University, Pocheon-si, Gyeonggi-do, Republic of Korea, daejin.ac.kr; ^4^ College of Nursing Science, Kyung Hee University, Seoul, Republic of Korea, khu.ac.kr

**Keywords:** death, emotional intelligence, end-of-life care, nurse, perception, performance, public hospital, stress

## Abstract

**Aims:**

To examine the relationships among end‐of‐life care stress, perception of a good death, emotional intelligence, and end‐of‐life care performance and the factors influencing end‐of‐life care performance of public hospital nurses.

**Background:**

Korean society is entering a superaging phase, and with growing social interest in end‐of‐life health, the need for end‐of‐life care is steadily increasing. End‐of‐life care stress, perceptions of a good death, emotional intelligence, and end‐of‐life care performance are interrelated, and comprehensive research that applies these variables is needed.

**Methods:**

A cross‐sectional descriptive design was employed and adheres to the STROBE guidelines. Participants were 146 nurses caring for terminally ill patients in a public hospital, South Korea. Measures were the general and job‐related characteristics list, the end‐of‐life care stress scale, the Well‐Dying Awareness Scale (WDAS), Wong and Law Emotional Intelligence Scale (WLEIS), and end‐of‐life care performance scale. Data were collected from February to April, 2025, and were analyzed using SPSS/WIN 29.0 program.

**Results:**

A positive correlation emerged between perception of a good death and end‐of‐life care performance (*r* = 0.19, *p* = 0.019). Department, end‐of‐life care education, and perception of a good death were statistically significant factors influencing end‐of‐life care performance. The explanatory power of the regression model was 12.1% (Adj. *R* ^2^ = 0.121).

**Conclusions:**

Department, end‐of‐life care education, and perception of a good death were significant factors associated with end‐of‐life care performance of public hospital nurses.

**Implications for Nursing Management:**

Expanding access to end‐of‐life care education and fostering a positive perception of good death among public hospital nurses are important strategies for improving end‐of‐life care performance. Nurse managers should ensure equitable access to end‐of‐life care education across all departments and create supportive organizational environments that encourage nurses to reflect on death‐related experiences.

## 1. Introduction

Advancements in medical technology continue to play a significant role in the promotion of human health and the maintenance of life. While these technologies are primarily used for treatment, they are also sometimes used to prolong the process of death. South Korea responded to the growing need for decision‐making regarding life‐sustaining treatment by enacting, in February 2016, legislation formally titled the “Act on Hospice, Palliative Care, and Decisions on Life‐Sustaining Treatment for Patients at the End of Life” [1]. Since August 2024, general hospitals and nursing hospitals with 300 or more beds are required to maintain at least one designated end‐of‐life room to ensure that patients experience a dignified end‐of‐life process. In conjunction with the growing social interest in end‐of‐life health, the demand for end‐of‐life and palliative care has increased globally and in South Korea [2, 3]. In this context, nurses are required to take on more active roles in coordinating and delivering end‐of‐life care, which may expand their responsibilities and workload. This, in turn, may contribute to increased physical and emotional burden among nurses [4, 5].

In particular, public hospital nurses serve as the primary caregivers for terminally ill patients, and they are responsible for supporting them in maintaining a positive outlook on life as the patients confront death [4]. Although medical interventions may no longer be effective, nurses caring for terminally ill patients are obligated to deliver adequate care that enables patients to preserve their human dignity and face their final moments with peace [4, 6, 7]. Nevertheless, nurses providing end‐of‐life care encounter profound inner challenges and experience high levels of work‐related stress stemming from patients’ and their guardians’ unrealistic expectations for a cure, recurring idealized hopes, and the subsequent disconnect with reality [8, 9]. Moreover, nurses providing end‐of‐life care experience increased physical and psychological stress without sufficient time for rest and recovery [4, 5, 10]. In the midst of patients struggling with the reality of death and their families who must send them off, nurses often endure loneliness and depression, accompanied by intense fear and anxiety related to death, often leading them to avoid confronting the patient’s death [5, 8–10]. Therefore, an attitude that acknowledges the concept of a good death enables nurses providing end‐of‐life care to adaptively explore suitable alternatives even in stressful situations and promptly administer the necessary end‐of‐life care. In other words, establishing a proper understanding of a good death can significantly increase nurses’ opportunities to provide high‐quality care to terminally ill patients. In particular, nurses’ insight into the concept of a good death serves to enhance both the spiritual well‐being of terminally ill patients and the psychological stability of their family members. Ultimately, for nurses, acknowledging the concept of a good death serves as a means to efficiently achieve the goals of end‐of‐life care [11, 12].

In addition, due to the death, suffering, and medical limitations of terminally ill patients, the grief experienced by their families has been shown to significantly influence the physical and mental health of nurses providing end‐of‐life care. Therefore, emotional intelligence has been recognized as a valuable resource that enables nurses to achieve more stable inner healing, even when faced with stressful situations involving terminally ill patients [13]. Emotional intelligence is essential in establishing a seamless therapeutic relationship between terminally ill patients and healthcare professionals, constituting the foundation for providing high‐quality medical services. Furthermore, nurses’ appropriate emotional intelligence allows them to calmly and realistically evaluate circumstances amid stressful and impulsive situations [14, 15].

Nurses’ stress, emotional intelligence, and perception of a good death, as discussed above, are important modifiable factors from a nursing management perspective. Specifically, adequate staffing and organizational support can buffer nurses’ end‐of‐life care stress [16], while leadership that provides structured education and emotional support contributes to the enhancement of emotional intelligence [17, 18]. In addition, a stable work environment and organizational education systems may influence nurses’ perception of a good death [19]. Therefore, these variables can be understood as modifiable through organizational and managerial strategies and should be considered key factors influencing nurses’ cognitive appraisal and coping processes in end‐of‐life care situations, ultimately affecting end‐of‐life care performance.

From a theoretical perspective, these variables can be explained based on Lazarus and Folkman’s transactional model of stress and coping [20], which posits that individuals respond to stressful situations through cognitive appraisal and coping processes, ultimately influencing adaptation outcomes. In the context of end‐of‐life care, emotional intelligence may function as a coping resource that enables nurses to regulate their emotions and respond effectively to stressful situations. In addition, perception of a good death may influence nurses’ cognitive appraisal and attitudes toward end‐of‐life situations, facilitating more positive interpretations and adaptive responses. Through these processes, nurses may cope more effectively with end‐of‐life care situations, which is expected to contribute to improved end‐of‐life care performance.

As previously discussed, stress related to end‐of‐life care, perception of a good death, and emotional intelligence are key factors affecting end‐of‐life care performance; however, studies that both apply and comprehensively analyze these variables have been limited. In particular, previous studies on end‐of‐life care have primarily focused on nurses working in intensive care units (ICUs), with the majority addressing end‐of‐life care stress, attitudes toward withdrawal of life‐sustaining treatment, and moral distress [21, 22], which may limit the generalizability of these findings to public hospital nurses. Public hospitals often serve socioeconomically vulnerable populations and operate under resource constraints compared to private hospitals, reflecting differences in patient composition and organizational contexts [23, 24]. Similarly, in South Korea, public hospital users are more likely to be older, have low income, and have chronic conditions, whereas private hospitals are more commonly utilized by individuals with a higher socioeconomic status [25]. These differences may influence nurses’ work environments and care performance. Therefore, this study aims to examine end‐of‐life care stress, perception of a good death, and emotional intelligence in relation to care performance among nurses working in public hospital settings, providing foundational data to develop strategies that enhance care for terminally ill patients while alleviating nurses’ work‐related stress.

The purpose of this study was to examine the relationships among end‐of‐life care stress, perception of a good death, emotional intelligence, and end‐of‐life care performance and the factors influencing end‐of‐life care performance of public hospital nurses. Accordingly, the study addressed the following research questions:

(1) What are the relationships between end‐of‐life care stress, perception of a good death, and emotional intelligence and end‐of‐life care performance?
(2) Which factors significantly influence end‐of‐life care performance among public hospital nurses?

## 2. Methods

### 2.1. Design, Sample, and Settings

This study employed a cross‐sectional descriptive design, adhering to the STROBE guidelines. Participants were nurses employed at a public hospital who met the following inclusion criteria: at least 6 months of work experience in a public hospital, prior experience in end‐of‐life care, and a demonstrated understanding of the study purpose with voluntary consent to participate. Nurses lacking prior end‐of‐life care experience in a public hospital setting were excluded. Sample size was determined using G∗Power 3.1.9 [26], based on an effect size of 0.15, a significance level of 0.05, and a power of 0.80, yielding a minimum required sample of 139. To allow for an anticipated 10% attrition rate, questionnaires were distributed to 155 nurses; 153 were returned (return rate = 98.7%), and 146 complete responses were retained for analysis after excluding 7 incomplete questionnaires.

### 2.2. Instrumentation

Participants’ general and job‐related characteristics were assessed using eight items: gender, age, educational level, total clinical experience, department, current position, prior end‐of‐life experience involving family or others, and end‐of‐life care education.

End‐of‐life care stress was measured using the scale developed by Lee [27], comprising 40 items across seven domains: negative attitudes of patients and caregivers (8 items), difficulty allocating time to terminally ill patients (7 items), care burden (7 items), excessive workload (5 items), interpersonal conflict (6 items), insufficient expertise (3 items), and conflicts over medical limitations (4 items). Items were rated on a 5‐point Likert scale, with higher scores reflecting greater stress. Internal consistency was *α* = 0.93 at development [27] and *α* = 0.94 in this sample.

Perception of a good death was measured with the Well‐Dying Awareness Scale (WDAS; [28]), 23‐item instrument spanning six domains: social (6 items), psychological (6 items), autonomous (3 items), physical (3 items), spiritual (2 items), and independent well‐dying (3 items). Each item was scored on a 5‐point Likert scale, with higher totals indicating a stronger perception of a good death. Cronbach’s *α* was 0.93 in the original validation [28] and 0.90 in the current study.

Emotional intelligence was assessed using the Wong and Law Emotional Intelligence Scale (WLEIS [29]), in its Korean‐language adaptation by Lee and Park [30]. The 16‐item measure covers four subdimensions—self‐emotion appraisal, others’ emotion appraisal, regulation of emotion, and use of emotion—each represented by four items rated on a 7‐point Likert scale, where higher scores denote greater emotional intelligence. Reliability was *α* = 0.87 in both the original [29] and Korean validation studies [30], and *α* = 0.84 in the present sample.

End‐of‐life care performance was evaluated using the instrument developed by Park and Choi [31], consisting of 22 items distributed across three domains: physical (8 items), psychological (8 items), and spiritual (6 items). Responses were rated on a 4‐point Likert scale, with higher scores reflecting better performance. Cronbach’s *α* was 0.93 at development [31] and 0.91 in this study.

### 2.3. Ethical Considerations

This study received approval from the Institutional Review Board of K University (KHSIRB‐24‐715). Nurses were recruited through QR‐coded posters displayed in nursing stations across departments, enabling them to access the survey on a voluntary and independent basis; this approach was intended to reduce potential coercion arising from the hospital’s hierarchical structure by avoiding direct recruitment through hospital leadership.

Prior to participation, participants provided electronic informed consent. They were informed of the study’s purpose, procedures, confidentiality, and anonymity, as well as their right to withdraw at any point without penalty, before completing the survey. Consent was confirmed via an online checkbox prior to granting access to the questionnaire. Responses were anonymized through coded identifiers, and data will be retained securely for 3 years in line with the Korean Bioethics and Safety Act, after which it will be permanently deleted.

### 2.4. Data Collection

Following IRB approval, the researcher obtained cooperation from the nursing department at a single public hospital in South Korea. Recruitment notices with study details and QR codes were displayed in nursing stations across departments, and data were collected between February 20 and April 13, 2025. Participants completed the questionnaire online via URL or QR code, with an estimated completion time of 15 min.

### 2.5. Data Analysis

Data were analyzed using SPSS/WIN 29.0 (IBM Corp., Armonk, NY, USA). General and job‐related characteristics of participants were summarized using frequencies, percentages, means, and standard deviations, and the same descriptive statistics were applied to characterize levels of end‐of‐life care stress, perception of a good death, emotional intelligence, and end‐of‐life care performance. Group differences in these four variables according to participants’ general and job‐related characteristics were examined using *t*‐tests and one‐way ANOVA, with Scheffe post hoc comparisons. Associations among end‐of‐life care stress, perception of a good death, emotional intelligence, and end‐of‐life care performance were assessed using Pearson’s correlation coefficients, and multiple regression analysis was conducted to identify factors influencing end‐of‐life care performance.

## 3. Results

General and job‐related characteristics of study participants are shown in Table 1. This study included 146 public hospital nurses, of whom 10 (6.8%) were male and 136 (93.2%) were female. Among the participants, 83 (56.8%) were aged 30–39, with an average age of 31.59. The majority of nurses (116, 79.4%) had graduated from college. Regarding clinical experience, 70 (47.9%) nurses had 6–10 years of clinical experience, followed by 40 (27.4%) nurses with 5 years or less, and 36 (24.7%) nurses with 11 or more years, resulting in an average clinical experience of 7.93 years. Most participants worked in non‐ICU departments (78.1%), whereas 21.9% worked in ICUs. In terms of position, the majority of nurses (89.7%) were staff nurses. Additionally, 103 (70.5%) public hospital nurses answered that they had experienced the death of a family member or acquaintance and 77 (52.7%) nurses answered that they had received end‐of‐life care training (Table 1).

**TABLE 1:** General and job‐related characteristics of the study participants (*n* = 146).

Variables	*N*	%	Mean (SD)
Gender			
Male	10	6.8	
Female	136	93.2	
Age (year)			
20–29	56	38.4	
30–39	83	56.8	31.59 (4.54)
40–49	7	4.8	
Educational level			
College	8	5.5	
University	116	79.4	
Master	22	15.1	
Total clinical experience (year)			
5≥	40	27.4	
6–10	70	47.9	7.93 (4.53)
≥ 11	36	24.7	
Department			
Non‐ICU^†^	114	78.1	
ICU	32	21.9	
Current position			
Staff nurse	131	89.7	
Managerial position^††^	15	10.3	
End‐of‐life experiences of family or others			
Yes	103	70.5	
No	43	29.5	
End‐of‐life care education			
Yes	77	52.7	
No	69	47.3	

^†^Non‐ICU departments included medical wards, surgical wards, and other general units.

^††^Managerial positions included charge nurses and head nurses.

The mean score for end‐of‐life care stress was 3.68 (SD = 0.52) out of 5, indicating a moderately high level. The mean score for perception of a good death was 3.75 (SD = 0.51) out of 5, also reflecting a moderately high level. The mean emotional intelligence score was 4.01 (SD = 0.79) out of 7, indicating a moderate level. The mean score for end‐of‐life care performance was 2.51 (SD = 0.45) out of 4, also indicating a moderate level.

Differences in end‐of‐life care stress, perception of a good death, emotional intelligence, and end‐of‐life care performance according to the general and job‐related characteristics of study participants are shown in Table 2. End‐of‐life care stress was significantly higher among female nurses than male nurses (*t* = −2.07, *p* = 0.040). The perception of a good death varied significantly according to the educational level (*F* = 5.83, *p* = 0.013), with post hoc analysis revealing that nurses with a master’s degree or higher demonstrated a significantly higher perception of a good death compared to those with a college degree. The perception of a good death was also found to be statistically significant among those who had experienced the death of a family member or acquaintance (*t* = 2.80, *p* = 0.003). Regarding end‐of‐life care performance, nurses working in the ICU demonstrated significantly higher performance than those in non‐ICU departments (*t* = −3.47, *p* = 0.001). All other general and job‐related characteristics were not significantly associated with any of the study variables (Table 2).

**TABLE 2:** Differences in end‐of‐life care stress, perception of a good death, emotional intelligence, and end‐of‐life care performance according to the general and job‐related characteristics of public hospital nurses (*n* = 146).

Variables	End‐of‐life care stress		Perception of a good death		Emotional intelligence		End‐of‐life care performance	
	Mean (SD)	*t* or *F* (*p*) *Scheffe*	Mean (SD)	*t* or *F* (*p*) *Scheffe*	Mean (SD)	*t* or *F* (*p*) *Scheffe*	Mean (SD)	*t* or *F* (*p*) *Scheffe*
Gender								
Male	3.36 (0.55)	−2.07 (0.040^∗^)	3.73 (0.39)	−0.13 (0.90)	3.56 (0.83)	−1.92 (0.057)	2.73 (0.51)	1.62 (0.108)
Female	3.70 (0.51)		3.76 (0.52)		4.05 (0.78)		2.49 (0.44)	
Age (year)								
20–29	3.68 (0.44)	0.52 (0.603)	3.71 (0.50)	0.39 (0.678)	3.94 (0.78)	0.82 (0.444)	2.49 (0.47)	2.37 (0.097)
30–39	3.70 (0.54)		3.78 (0.53)		4.08 (0.80)		2.48 (0.43)	
40–49	3.38 (0.80)		3.80 (0.45)		3.79 (0.71)		2.86 (0.49)	
Educational level								
College^a^	3.70 (0.53)	1.59 (0.207)	3.78 (0.93)	5.83 (0.013^∗^) *c* > *b*	4.25 (0.79)	1.02 (0.364)	2.35 (0.38)	2.81 (0.063)
University^b^	3.67 (0.51)		3.69 (0.46)		3.97 (0.73)		2.48 (0.43)	
Master^c^	3.72 (0.62)		4.07 (0.47)		4.17 (1.05)		2.70 (0.54)	
Total clinical experience (year)								
5 ≥	3.59 (0.49)	1.37 (0.259)	3.68 (0.53)	0.72 (0.490)	3.97 (0.92)	0.077 (0.259)	2.54 (0.47)	0.27 (0.767)
6–10	3.75 (0.50)		3.80 (0.50)		4.03 (0.78)		2.50 (0.45)	
≥ 11	3.63 (0.58)		3.76 (0.51)		4.02 (0.64)		2.47 (0.43)	
Department								
Non‐ICU	3.73 (0.50)	2.14 (0.034^∗^)	3.71 (0.52)	−1.86 (0.066)	3.96 (0.72)	−1.52 (0.132)	2.44 (0.40)	−3.47 (0.001^∗∗^)
ICU	3.50 (0.55)		3.90 (0.46)		4.20 (0.97)		2.74 (0.53)	
Current position								
Staff nurse	3.67 (0.53)	−0.49 (0.624)	3.71 (0.49)	−2.65 (0.009)	4.00 (0.78)	−0.51 (0.61)	2.48 (0.45)	−1.92 (0.057)
Managerial position	3.74 (0.45)		4.07 (0.61)		4.11 (0.85)		2.71 (0.40)	
End‐of‐life experiences of family or others								
Yes	3.71 (0.55)	1.25 (0.212)	3.83 (0.50)	2.80 (0.003^∗∗^)	3.97 (0.78)	−1.15 (0.252)	2.53 (0.50)	1.01 (0.403)
No	3.60 (0.42)		3.57 (0.50)		4.13 (0.79)		2.44 (0.43)	
End‐of‐life care education								
Yes	3.67 (0.53)	−0.20 (0.845)	3.73 (0.52)	−0.71 (0.478)	3.91 (0.79)	−1.70 (0.092)	2.57 (0.39)	1.87 (0.064)
No	3.69 (0.51)		3.79 (0.50)		4.13 (0.77)		2.43 (0.50)	

*Note:* The a, b, and c mean the result of Scheffe post hoc test.

^∗^ *p* < 0.05.

^∗∗^ *p* < 0.01.

Regarding the correlations among the study variables, end‐of‐life care stress was positively correlated with perception of a good death (*r* = 0.23, *p* = 0.005), and perception of a good death was positively correlated with end‐of‐life care performance (*r* = 0.19, *p* = 0.019). Emotional intelligence showed no significant correlations with the other study variables.

Factors influencing end‐of‐life care performance are presented in Table 3. Prior to conducting the multiple regression analysis, the assumptions of the regression model were examined. The residual plot indicated homoscedasticity, and the Durbin–Watson statistic was 2.16, indicating independence of residuals. In addition, the tolerance values ranged from 0.80 to 0.98, and the variance inflation factor (VIF) values ranged from 1.02 to 1.26, indicating no multicollinearity among the independent variables. The regression analysis showed that department (*β* = 0.28, *p* = 0.002), end‐of‐life care education (*β* = 0.23, *p* = 0.005), and perception of a good death (*β* = 0.17, *p* = 0.046) were significant factors influencing end‐of‐life care performance. Specifically, nurses working in ICUs and those who had received end‐of‐life care education demonstrated higher end‐of‐life care performance.

**TABLE 3:** Factors influencing end‐of‐life care performance (*n* = 146).

Variables	*B*	SE	*β*	*t*	*p*	Adj *R* ^2^	*F* (*p*)
(Constant)	1.50	0.39		3.82	< 0.001		
Total clinical experience	−0.03	0.049	−0.05	−0.64	0.521	0.121	3.864 (0.001)
Department	0.30	0.10	0.28	3.16	0.002^∗∗^		
Current position	0.03	0.13	0.23	0.27	0.789		
End‐of‐life care education	0.21	0.07	0.23	2.83	0.005^∗∗^		
End‐of‐life care stress	0.01	0.07	0.01	0.14	0.888		
Perception of a good death	0.15	0.07	0.17	2.02	0.046^∗^		
Emotional intelligence	0.07	0.05	0.12	1.47	0.143		

*Note:* Reference groups for dummy variables were ≤ 5 years of clinical experience, non‐ICU department, staff nurse position, and no end‐of‐life care education.

Abbreviation: SE, standard error.

Adj. *R* ^2^ = adjusted *R*‐squared.

^∗^ *p* < 0.05.

^∗∗^ *p* < 0.01.

In addition, nurses with a stronger perception of a good death showed better end‐of‐life care performance. This regression model accounted for 12.1% of the variance (Adj. *R* ^2^ = 0.121, *F* = 3.864, *p* = 0.001). The adjusted *R* ^2^ of this study was 0.121, which approaches a medium level of explanatory power (*f* ^2^  = 0.138) according to Cohen’s [32] criteria. Ozili [33] argued that an *R* ^2^ of 0.10 or above is acceptable in social science research under the condition that some or most of the predictor variables are statistically significant, and that a low *R* ^2^ does not indicate a flaw in the model given that the purpose of social science modeling is not to fully predict human behavior but to examine whether specific variables have a significant effect on the dependent variable.

## 4. Discussion

The majority of public hospital nurses who participated in this study were staff nurses under the age of 39 with less than 10 years of clinical experience. In terms of differences in study variables according to general and job‐related characteristics, end‐of‐life care stress was significantly higher among female nurses than male nurses. However, this finding should be interpreted with caution given that only 8 male nurses participated in this study, which limits the generalizability of gender‐based comparisons. Regarding end‐of‐life care performance, ICU nurses demonstrated significantly higher performance than non‐ICU nurses. This finding is consistent with Price et al. [34], who reported significantly higher perceived competency in palliative and end‐of‐life care among ICU nurses compared to nurses in other inpatient settings. This may be attributed to ICU nurses’ more frequent exposure to dying patients and greater experience with end‐of‐life situations in clinical practice. These findings suggest that targeted educational interventions are needed, particularly for non‐ICU nurses, to strengthen their end‐of‐life care competencies and prepare them for the unique challenges of caring for terminally ill patients in public hospital settings.

In this study, nurses with a master’s degree or higher demonstrated a significantly higher perception of a good death compared to those with a college degree. This is consistent with previous research suggesting that higher educational attainment is associated with more favorable perceptions of death and dying among healthcare professionals [35] and suggests that advanced education may foster deeper reflection on death and dying. Additionally, nurses who had experienced the death of a family member or acquaintance showed a significantly higher perception of a good death.

The significant predictors of end‐of‐life care performance among public hospital nurses were department, end‐of‐life care education, and perception of a good death. Among these, department was the strongest predictor, with ICU nurses demonstrating significantly higher performance than non‐ICU nurses. This finding is consistent with Price et al. [34], who reported significantly higher perceived competency in end‐of‐life care among ICU nurses compared to those in other inpatient settings. Given that 78.1% of the participants in this study were non‐ICU nurses, this finding carries important implications. Non‐ICU nurses may have less frequent exposure to dying patients, which may limit opportunities to develop end‐of‐life care competencies. Nurse managers should, therefore, recognize this gap and actively support non‐ICU nurses by facilitating collaboration with palliative care teams and creating an environment where end‐of‐life care is treated as a shared responsibility across all departments, not only within the ICU.

End‐of‐life care education was also a significant predictor of performance. Despite its demonstrated importance, only 52.7% of the participants had received end‐of‐life care education, suggesting that nearly half of public hospital nurses lacked this foundational preparation. This aligns with Ghaemizade Shushtari et al. [36], who demonstrated that structured end‐of‐life care education significantly improved nurses’ knowledge, attitudes, and performance. These findings highlight the urgent need to expand access to end‐of‐life care education in public hospital settings. End‐of‐life care content should be incorporated into orientation programs for newly hired nurses, and regular in‐service education should be provided on an ongoing basis. Nurse managers play a key role in enabling this by adjusting work schedules to ensure that all nurses, regardless of department or position, have equitable opportunities to participate in education.

Perception of a good death was also a significant predictor of end‐of‐life care performance, indicating that nurses who held a more positive perception of good death demonstrated higher end‐of‐life care performance. This is consistent with Park et al. [37], who found that positive perceptions of death were significantly associated with end‐of‐life care performance, mediated by nurses’ attitudes toward end‐of‐life care. These findings suggest that fostering a positive perception of good death is not only an educational goal but also an organizational one. Educational programs that encourage nurses to reflect on the meaning of death and explore their personal values around dying should be systematically developed. At the same time, nurse managers can support this process by creating a culture in which nurses feel comfortable discussing death‐related experiences, for example, through regular debriefing sessions and case conferences following end‐of‐life care situations.

In this study, emotional intelligence was not a significant predictor of end‐of‐life care performance. This finding differs from the theoretical assumption that emotional intelligence facilitates effective coping and interpersonal relationships in nursing practice. One possible explanation is that emotional intelligence may influence end‐of‐life care performance indirectly through nurses’ perceptions and coping processes rather than exerting a direct effect. In addition, the emotional intelligence scale used in this study was a self‐report measure, which may not fully reflect nurses’ actual emotional responses and competencies in end‐of‐life care situations. Furthermore, organizational factors such as department characteristics and access to end‐of‐life care education may have had a stronger influence on end‐of‐life care performance than individual emotional characteristics in the public hospital setting. Future research is needed to further examine the pathways through which emotional intelligence influences end‐of‐life care performance.

This study contributes to the growing body of literature on end‐of‐life care nursing by examining the combined influence of end‐of‐life care stress, perception of a good death, and emotional intelligence on end‐of‐life care performance among public hospital nurses in South Korea—a context that has received limited attention in the international literature. While previous studies have largely focused on hospice or ICU settings, this study highlights the unique challenges faced by nurses in public hospital environments that serve socioeconomically vulnerable populations. The findings identify department, end‐of‐life care education, and perception of a good death as significant predictors of end‐of‐life care performance, offering concrete and actionable evidence for nursing practice. Specifically, the results suggest that expanding access to end‐of‐life care education for non‐ICU nurses and cultivating a positive perception of good death through reflective practice and supportive organizational cultures are promising strategies for improving the quality of end‐of‐life care in public hospital settings.

### 4.1. Study Limitations

This study was conducted among nurses working at a single public hospital in South Korea. Therefore, caution is needed when generalizing the findings to nurses working in other public hospital settings or healthcare institutions. In addition, because this study used a cross‐sectional descriptive design, causal relationships among the study variables could not be established. Furthermore, because this study relied on self‐reported quantitative questionnaires, there may be limitations in fully capturing the complex and nuanced experiences related to end‐of‐life care. Future studies using qualitative or mixed‐method approaches are recommended to gain a deeper understanding of nurses’ experiences and perceptions regarding end‐of‐life care.

## 5. Conclusions

In conclusion, department was the strongest predictor of end‐of‐life care performance among public hospital nurses, followed by end‐of‐life care education and perception of a good death. The results of this study are significant as they identified factors influencing end‐of‐life care performance by analyzing the correlations among end‐of‐life care stress, perception of a good death, emotional intelligence, and end‐of‐life care performance among public hospital nurses in South Korea.

Furthermore, as societies age rapidly and chronic, complex conditions become more prevalent, demand for palliative and end‐of‐life care continues to rise accordingly [38]. This trend has highlighted the expanding role of the public health sector in addressing the needs of patients with life‐limiting illnesses [39]. Public hospitals, which disproportionately serve socioeconomically vulnerable and medically underserved populations [23–25], play a central role in delivering such care. As the primary caregivers in these settings, nurses are at the forefront of end‐of‐life care delivery [40], and the growing demand associated with an aging population directly increases their exposure to end‐of‐life situations and greater clinical and emotional demands. In this context, understanding the factors that influence end‐of‐life care performance among public hospital nurses is particularly important, and these findings provide foundational evidence for improving the quality of end‐of‐life care in such settings.

## 6. Implication for Nursing Management

Based on the results of this study, it is necessary to implement training programs that will enhance the perception of a good death and strengthen end‐of‐life care competencies in order to achieve high‐quality nursing performance among nurses providing end‐of‐life care in public hospitals. In particular, ward managers in public hospitals should pay close attention to the key factors influencing end‐of‐life care identified in this study to enhance nurses’ end‐of‐life care performance. The results of this study can also be utilized in developing nursing strategies and systems aimed at enhancing end‐of‐life care performance among public hospital nurses. As such, future qualitative research is necessary to understand and analyze nurses’ acceptance of end‐of‐life care and their perceptions of death, while experimental studies are required to develop nursing intervention programs for nurses providing end‐of‐life care and to verify their effectiveness.

## Author Contributions

Saekyae Shin: conceptualization, methodology, investigation, formal analysis, validation, visualization, writing–original draft preparation, and writing–review and editing. Mi Gyoung Kim: conceptualization, data curation, methodology, validation, and writing–original draft preparation. Minkyung Gu: conceptualization, data curation, methodology, validation, and writing–original draft preparation. Sohyune Sok: conceptualization, methodology, investigation, formal analysis, validation, visualization, writing–original draft preparation, writing–review and editing, and supervision.

## Funding

No funding was received for this manuscript.

## Disclosure

All authors have read and approved the final manuscript.

## Ethics Statement

The study was approved by the Kyung Hee University Institutional Review Board (No. KHSIRB‐24‐715).

## Conflicts of Interest

The authors declare no conflicts of interest.

## Data Availability

The data that support the findings of this study are available from the corresponding author upon reasonable request.
